# Personalized Medicine in Acute Care Surgery: Are We Ready to Deal with Our Failures?

**DOI:** 10.3390/jpm12081322

**Published:** 2022-08-17

**Authors:** Roberto Bini, Michele Altomare

**Affiliations:** 1 Department of Emergency Care, General Surgery and Trauma Team, Azienda Socio Sanitaria Territoriale Grande Ospedale Metropolitano Niguarda, 20162 Milano, Italy; 2 Department of Surgical Sciences, Sapienza University of Rome, Piazzale Aldo Moro 5, 00185 Rome, Italy

During the first decade of the 21st century, the American College of Surgeons Committee on Trauma (ACS—COT), the Western Trauma Association (WTA), the Eastern Association for the Surgery of Trauma (EAST), and the American Association for the Surgery of Trauma (AAST) established an ad hoc committee to develop a new specialty that embraces trauma surgery, critical surgical care, and emergency surgery [1,2]. This new entity was called Acute Care Surgery (ACS). In the USA, many programs were built for attending surgeons to offer the possibility of acquiring the fundamental multidisciplinary skills needed. This specialty was created in response to the need to have surgeons trained to handle surgical emergencies and responding to what William S. Halsted (1852–1922) predicted during the last century “*Every important hospital should have on its resident staff of surgeons at least one who is well and able to deal with any emergency that may arise*” [3]. Training programs for these new specialists are pretty heterogeneous in different countries.

Although the fundamental pillars of ACS are urgent conditions, specialists are trained through the practice of elective surgery. This could allow the acquisition of the absolute basics of anatomy and surgical technique. One of the specific aspects of an acute care surgeon is dealing with rescue surgery [4] on a daily basis, defined as the ability to manage postoperative and post-procedural complications. This aspect requires both the skills of individual practitioners and the resources and commitment of entire institutions [5]. Patients who develop a postoperative complication are at increased risk of adverse outcomes. There are many factors affecting the risk of developing complications in surgical patients. In a review, De Vries et al. [6] reported that the overall median incidence of in-hospital adverse events was 9.2%. Authors showed that more than half (56.3%) of patients experienced no or minor disability, whereas 7.4% of events were lethal. Operation-related events constituted the majority of reported adverse events (39.6%). Adverse events during hospital admission affect nearly one out of ten patients [6].

Mc Coy et al. [7] reported that emergency operations accounted for 14.6% of general surgery procedures but 53.5% of all postoperative deaths. Infection of the incisional surgical site and pneumonia were the more frequent complications, whereas stroke, major bleeding, myocardial infarction, and pneumonia exhibited the strongest associations with postoperative death. One only needs to consider that appendicitis, cholecystitis, and bowel obstruction result in a number of complications in terms of medical or surgical care to better understand the magnitude of the phenomenon. These patients need immediate intervention to rescue them from a possible significant complication.

Ghaferi AA et al. [8] pointed out that the number of complications in high-volume hospitals is not so different from that in low-volume hospitals. Still, the real difference lies in the ability to perform rescue surgery, which is better in high-volume hospitals. Many of these patients require relaparotomy, some will need intensive care, and a minority will need interventional radiology. The expertise required of an acute care surgeon should be sufficient to ensure the best decision-making process in the least amount of time [4]. Therefore, the system’s inability to best deal with a complication resulting in patient death is called Failure to Rescue (FTR). In the literature, the incidence of FTR varies between 8.0% and 16.9%. FTR was found to be inversely related to hospital volume and nurse staffing levels. The delayed escalation of intensity of care occurred in 20.7–47.1% of patients, and higher mortality was observed in this group. Causes of delayed escalation include mainly the inability to make an early diagnosis and communication failures [9]. FTR is now considered a valid parameter for judging the quality of a system, and its improvement goes hand-in-hand with system upgrading. Some factors leading to FTR are related to patient characteristics, including frailty, congestive heart failure (CHF), renal failure, and serum albumin. In contrast, others are system-related, such as a favorable nurse-patient ratio, highly trained staff in a closed environment, and multidisciplinary teams. In reality, not all hospitals have the resources to implement all of these factors. The importance of team communication and the ability to recognize and escalate care for a patient can differentiate a failure from a huge and unhoped rescue [9]. According to the authors, the combined use of early warning scoring systems (such as the National Early Warning Score (NEWS) and the Modified Early Warning Score (MEWS)) and organized training programs to promote communication among team elements could lead to a reduction in FTR [10].

The most important characteristics of patients referred for surgery are advanced age and comorbidities, which often coexist in the same patient; for this reason, data on the role of frailty and predictive scores to measure its impact are increasingly found in the literature. Kennedy et al. reported that the prevalence of frailty amongst patients undergoing emergency abdominal surgery (EGS) was 30.8.% and the mortality rate amongst the frail undergoing EGS was 24.7%. The defect was associated with an increased mortality rate compared with non-frail patients (OR 4.3, 95% CI 2.25–8.19%, *p* < 0.05, I^2^ = 80%). [11] Moreover, a meta-analysis demonstrated that frailty is a good marker for ultimately poor outcomes and may also be associated with prolonged hospital stay and the need for readmission [12]. The authors concluded that the frailty scoring system should be integrated into acute surgical assessment practice to aid decision making and the development of novel postoperative strategies [13]. A critical care surgeon’s significant contribution is to create a sole physician who deals simultaneously with the operating theatre, intensive care, and rescue surgery.

Moreover, critical care surgeons’ last but not most minor role is interacting with deteriorating in-ward surgical patients. To do so, they need to know and be familiar with scores that are predictive of postoperative complications or the reactivation of chronic disease. The Early Warning Score [14] is one of the most used and well-studied in Europe. More specific scores exist for each surgery branch to predict a worsening patient. Studies have shown that in approximately one-third of patients dying in-ward, heart rate and respiratory rate alterations are not detected as soon as they are presented [15]. For this reason, some authors have recently explored the potential role of the full-time monitoring of patients with low intensity of care or in the general ward; they suggest that even if the monitoring alone does not reduce the FTR rate, a daily clinical evaluation associated with score detection and full-time tracking could lead to a better outcome for surgical patients [16].

Being familiar with the signs that come with the early onset of postoperative complications is one of the principal skills that an acute care surgeon should develop during their training. The willingness to follow the patient’s journey from presentation in the emergency department to discharge is a core principle of Acute Care Surgery, which needs to be implemented in the next generation of surgeons. It is important to remember that deviations from the routine are always present in our patients. Only a real no-blame environment based on honest communication and mutual respect can focus the attention on what caused the problem, more than who caused it. To do so, we need to change centennial beliefs and behaviors, renovating our mental and practical approach to the work, relationships between colleagues, and the ability to deal with our failures.
